# Twitter (X) in Medicine: Friend or Foe to the Field of Interventional Cardiology?

**DOI:** 10.1016/j.jscai.2023.101136

**Published:** 2023-09-17

**Authors:** Mohammad Atif Rana, Grace Sorger, David A. Cox, George D. Dangas, Farshad Forouzandeh

**Affiliations:** a University Hospitals Harrington Heart & Vascular Institute, Case Western Reserve University School of Medicine, Cleveland, Ohio; b Allegheny College, Meadville, Pennsylvania; c Atrium Health Sanger Heart & Vascular Institute, Charlotte, North Carolina; d Zena and Michael A. Wiener Cardiovascular Institute, Icahn School of Medicine at Mount Sinai, New York, New York

**Keywords:** interventional cardiology, networking, social media, Twitter

## Abstract

Twitter, which recently changed its name to “X,” is a popular social media platform that is used widely across the world due to its easy accessibility through the internet. Due to more public familiarity, we referred to this social media platform as “Twitter (X)” in this article. Users can create posts, also called as “tweets” with a limitation of 280 characters and can add images, videos, and weblinks. While there are several social media platforms used by health care professionals, eg, Facebook, Instagram, Snapchat, TikTok, LinkedIn, and Periscope, Twitter (X) is an extremely popular platform among physicians, especially cardiologists. Due to its persistent growth and ever-expanding outreach, Twitter (X) is facilitating dissemination of scientific information including complex medical knowledge leading the way in collaborations, and in organizing and networking of various health care professionals, patients, and caregivers with shared medical interests. While there are several advantages of Twitter (X) as an important tool in our contemporary medical armamentarium, there are also some drawbacks. False information can be distributed effortlessly and can entrench unscientific beliefs as there is no peer-review process. Occasionally, discourteous discussions between health care professionals can be misinterpreted by users with limited medical knowledge. Other disadvantages include Health Insurance Portability and Accountability Act violations and hacking of accounts. Therefore, it is imperative for health care professionals interested to use this valuable tool to be familiar with and cautious about its potential risks and limitations.

## Highlights

• Twitter (X) is a very powerful social media platform in cardiology to improve outreach, education, and networking.
• Twitter (X) can be very useful in the timely dissemination of valuable information.
• It is prudent to consider and mitigate the risk of such a powerful tool in cardiology.
• Twitter (X) could disseminate misinformation, hacking, and oversimplification, and contribute to burnout.

## Central Illustration

**Figure:**
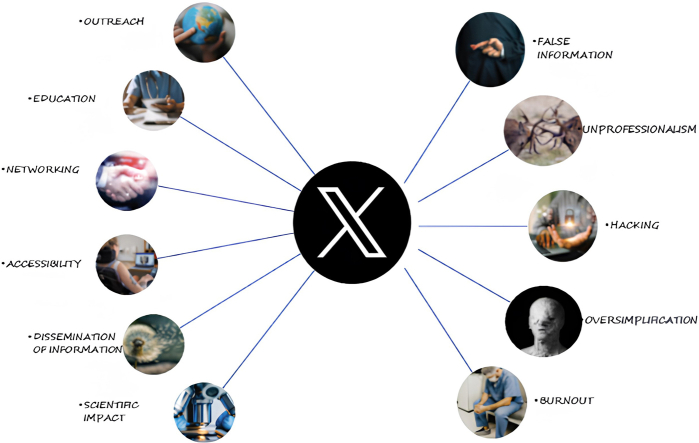

## Introduction

Social Media (SoMe) use is rapidly growing as an interactive communication tool among physicians and especially cardiologists.^1^, ^2^, ^3^, ^4^ While there are several SoMe platforms like Facebook, Instagram, YouTube, Snapchat, Periscope, LinkedIn, etc., Twitter (X) is one of the most used SoMe platforms by physicians.^5^, ^6^, ^7^, ^8^, ^9^, ^10^, ^11^, ^12^, ^13^ Twitter (X) is a microblogging website created in 2006 that allows users to create posts called “tweets” with a limit of 280 characters along with optional additional images, videos, web links, and links to other SoMe platforms.^14^^,^^15^ Users can utilize retweets and likes to endorse posts. Hashtags are promotional tools used in Twitter (X) to enhance and focus on the crux of a discussion and aid in identification of tweets marked with the same hashtag.^16^ Users have the choice to keep their account private or public.^17^ The American Medical Association (AMA) was the first organization to create a set of guidelines for SoMe use among health care professionals.^18^, ^19^, ^20^ More than 1000 cardiologists were estimated to be present on Twitter (X) as per a study from 2019.^21^ This number perhaps is significantly higher now.

## Benefits of Twitter (X) within cardiology

### Outreach

Since Twitter (X) is free and easily accessible through the internet, it enables virtual congregation of a diverse group of health care professionals, patients, and their caregivers spread throughout the world. ^2^ In those with internet access, there were no disparities in SoMe use, spread across race, ethnicity, and health status.^22^, ^23^ In a recent study by Guerra et al, among health care professionals from 35 countries, Twitter (X) (55.1%) was among the most widely used SoME platforms, only second to LinkedIn (60.8%).^24^ Furthermore, among the group of vascular interventional proceduralists, including vascular surgeons, interventional radiologists, and interventional cardiologists (ICs), ICs were most likely to be influencers and had the highest follower/following ratio.^25^ #CardioTwitter, which represents tweets related to cardiovascular sciences, has shown significant growth, with more than 600,000 tweets since October 2017.^26^ Twitter (X)’s major advantage over educational websites and online journals is the active interaction among a diverse and vast audience including medical professionals, patients, and caregivers.^27^

### Education

Every month thousands of cardiovascular medicine articles are published in various cardiology journals spread throughout the world in several languages. While this is the manifestation of the innovative spirit and scientific rigor of the cardiology community, it is not only difficult to access all these articles but also almost impossible to read through even a fraction of them. This gap is somewhat filled by SoMe applications like Twitter (X) where individual authors, clinicians, and health care workers can showcase and promote their work.

Furthermore, in the field of interventional cardiology, a procedure-based specialty, master experienced operators across the world share special techniques through SoME, which help solve difficult procedural issues, add to the interventional cardiology armamentarium, and propel the field forward.

Several ICs see and adopt these novel techniques, including at our institution, a large, university-based quaternary hospital in the USA, where one of our mid-career interventional cardiology attending learned the single-access technique for Impella (Abiomed)-assisted high-risk PCI through Twitter (X).^28^

One of the earliest uses of hashtags in cardiology was #RadialFirst, which was popularized by interventional cardiology leaders like Sunil Rao, MD. The number of tweets with #RadialFirst was 22,804 in 2017, 33,074 in 2018, 38,352 in 2019, and 9915 by April 2020.^29^ This not only popularized and helped in quicker adoption of radial access for coronary angiograms and interventions among ICs but also inspired other medical specialties like neurology/radiology to adopt radial access for neurointerventions (#RadialForNeuro).^29^

These instances have been regularly reported as noted recently in an article from *EuroIntervention*, where an IC learned about the left distal transradial approach (ldTRA) through Twitter (X)^30^^,^^31^ and very elegantly described how new interventional techniques, in their infancy, are now being presented through Twitter (X) leading to their promotion and subsequent adoption by ICs across borders and regions.

Similarly, use of axillary artery for large-bore access procedures and several techniques for complete total occlusion were popularized via Twitter (X).

Twitter (X) users can use hashtags to search for several topics, eg, #PercAx, #CTO101, and #ldTRA.

Since Twitter (X) is an interactive platform, physicians often “tag” specialty leaders and experts who usually promptly respond with their own comments if they choose to do so.

Twitter (X) facilitates access to various journal clubs (#ASEcho, #CardioNerds, #CardJC, etc.), webinars (#CSIvirtual, #TVTConnect, #ESCWebinar, etc.), and cardiovascular scientific sessions, and helps in the professional development of cardiologists.

Twitter (X) also promotes awareness among the general public as they have access to accounts of physician leaders. Various hashtags like #WorldHypertensionDay,^32^ #WorldThrombosisDay, #WorldHeartDay,^33^ and #ValveDiseaseDay have been used to raise awareness about heart conditions in the community. This was very pronounced during the COVID-19 pandemic when cardiology leaders regularly reached out to the general public through their own Twitter (X) accounts and national media Twitter (X) gave regular advice to the general public and emphasized the need for resources to timely manage patients with cardiac comorbidities.^34^

### Networking

Twitter (X) “tweets” can be used to announce information about upcoming meetings, propagate new medications and novel devices, advertise for research participant recruitment,^35^ as well as for new jobs and positions.

Several patients with rare medical and cardiovascular conditions like spontaneous coronary artery dissection, fibromuscular dysplasia, and heart transplant/left ventricular assist device recipients have developed patient-run networking groups on Twitter (X) to provide information regarding management, treatment processes, etc. which helps in allaying anxiety among the patients.

Health care workers, especially physicians and cardiologists, have utilized Twitter (X) to advocate against sexual and racial discrimination. Through use of hashtags like #WIC (Women in Cardiology), #SCAIWIN (Society for Cardiovascular Angiography & Interventions Women in Innovations), and #WomeninEP (Women in Electrophysiology), Twitter (X) has become a major platform for networking and community building.^36^

Academic cardiologists can pursue funding opportunities and collaborations with the global medical and research community through Twitter (X). Furthermore, tweets are increasingly being used for research through tweet mining using Twitter (X) application programming interface, NodeXL (Social Media Research Foundation), Topsy (Topsy), NCapture (Chrome extension), etc.^37^

### Accessibility

A recent survey of 485 physicians revealed daily use of SoMe by 24% of participants to explore medical information^38^ mostly due to easy access.

### Easy dissemination of information

Because of easy accessibility through mobile devices connected to the internet, Twitter (X) and other SoMe platforms are serving as an ingenious mode of dissemination of novel information across continents.^2^^,^^3^^,^^12^^,^^24^ Hashtags underscore pertinent discussion topics and facilitate searches by interested parties.^1^^,^^7^^,^^9^^,^^14^^,^^39^ Examples of such hashtags are #PercAx, #CTO101, #ldTRA, #WIC, #SCAIWIN, and #WomeninEP. Several educational conferences like Cardiovascular Innovations (#CVI2023), New Cardiovascular Horizons (#NCVH2024), and Society for Cardiovascular Angiography & Interventions (#SCAI), and cardiology journals like *Journal of American College of Cardiology* (#JACC) are easily searchable. By using such hashtags, educational conferences allow frequent posts during the conferences in real-time and give opportunities for better engagement of a larger audience.

Several society journals started using Twitter (X) within the past 2 decades, eg, *Catheterization and Cardiovascular Interventions*, the previous official journal of the Society for Cardiovascular Angiography & Interventions, in April 2011, *Circulation* of the American Heart Association in February 2012, journals of the American College of Cardiology in April 2015, and *EuroIntervention* and *European Heart Journal* of the European Society of Cardiology in September 2017. *JSCAI*, the current official journal of SCAI, started using Twitter (X) at the founding of the journal in January 2022.^40^

The general public often looks up SoMe platforms like Twitter (X) for public health communication and guidance regarding medical conditions. Tweets have been used by public health authorities for communication and have been analyzed to gauge public sentiment as well as to track and measure disease activity. This was pronounced during the COVID-19 pandemic^41^ and during spread of novel influenza A (H1N1).^42^

### Scientific impact

Due to effortless access, easy dissemination of information, and availability of experts, the Twitter (X) platform is well-poised to promote ideas and scientific literature. This is achieved through posts, hashtags, associated images, videos, and links added to tweets. A recent study by Sathianathen et al showed that attention received by publications on SoMe can predict future citations and scientific impact.^43^ Furthermore, Altmetrics, a broad group of metrics capturing the impact of a scientific paper measured by analyzing the number of views, discussions, saves, citations, and recommendations on various SoMe platforms (eg, Facebook, Twitter (X), Instagram, LinkedIn, Wikipedia, Pinterest, Reddit, YouTube, F1000Prime) and exported citations^40^ is increasingly being used as an alternative to traditional citations.

Tweets are being increasingly used to promote research papers with resultant increased outreach and engagement with new viewers.^2^^,^^3^^,^^44^

## Risks

### False information

Cardiovascular disease is the leading cause of death in the United States with 1 death every 34 seconds and 1 in 5 deaths overall.^45^ While published medical literature is always peer-reviewed and goes through the review process to get validated and approved, there is no such mechanism for medical information dissemination through SoMe where several influencers tweet information hidden behind the cloak of a free speech platform.^46^ Because of such (mis)information, some patients may refuse using medications or following medical advice that is based on rigorous research studies and careful approval processes such as well-known medications like statins. Similar disinformation is apparent around vaccinations as well which have led to significant morbidity and mortality as seen most recently in the COVID-19 pandemic.^47^, ^48^, ^49^, ^50^, ^51^

### Unprofessionalism

Often, there are instances of unprofessional behavior and tribalism among several specialties like interventional cardiology and cardiothoracic surgery, recently noted after the publication of the 5-year results of the EXCEL trial (Evaluation of XIENCE Versus Coronary Artery Bypass Surgery for Effectiveness of Left Main Revascularization).^52^ Such discussions, readily available to the general population, can sometimes be a source of confusion among patients with limited medical knowledge and without proper understanding of complex topics. Also, due to absence of systematic censorship on Twitter (X) compounded with the ability to hide behind a cloak of anonymity, few health care professionals irresponsibly use SoMe to poke fun at patients, which discredits physician-patient relationships. Furthermore, Twitter (X) being in the public domain can be accessed by coworkers, superiors, and current and future patients and poses a risk based on the SoMe activity of the health care professional.^7^ Health Insurance Portability and Accountability Act (HIPAA) violations pose an additional risk on SoME platforms like Twitter (X) and can have grave repercussions because of breach of confidentiality.^46^^,^^53^^,^^54^

### Hacking

Since SoMe use is an internet-based activity, user accounts can be hacked and misused by creating posts with misinformation and can subsequently tarnish a health care professional’s name and damage his/her career.^55^^,^^56^

### Oversimplification

Due to easy accessibility, a large part of the general population seeks medical knowledge and research medical conditions on the internet and through SoMe platforms like Twitter (X).^16^ While Twitter (X) and other SoMe platforms offer value, they are not a substitute for professional medical advice. Furthermore, it’s extremely difficult to offer comprehensive medical advice in a 280-character limit tweet.

### Burnout

A recent *JAMA* study shows that burnout among physicians, residents in training, and advanced practice clinicians has jumped to record highs from 45% in 2019, 40% to 45% in early 2020, 50% in late 2020 to 60% in late 2021.^57^ In these circumstances, it needs to be evaluated whether prudent time management and prioritizing decompressing with family and enjoying hobbies over spending time on SoMe will mitigate health care worker exhaustion.

## Conclusion

SoMe, particularly Twitter (X), is growing exponentially within cardiology and medicine and is becoming more popular among ICs continuously. Twitter (X) is a useful tool to reach a diverse and broad audience, facilitate effortless and easily accessible education, allow networking opportunities, recruitment, and advertisement for research, and aid in “moving the needle” in medicine through discussions on new medical practices. Twitter (X) also helps in engagement of public and medical professionals and the scientific impact of published papers. However, there are still risks of using it, notably, unprofessionalism, potential HIPAA violations, and the spread of false information, which need to be considered while taking advantage of this very valuable tool in the medical field of our era.

In brief, Twitter (X) is a powerful composite platform that delivers tremendous value to health care providers as well as health care consumers. While any powerful tool can be used for progress and improvement, there is always a potential for deleterious effects and harmful use (Central Illustration). A logical solution to this menace can be use of appropriate resources to monitor and “fact-check” information and its source, as much as possible.

**Central Illustration:**
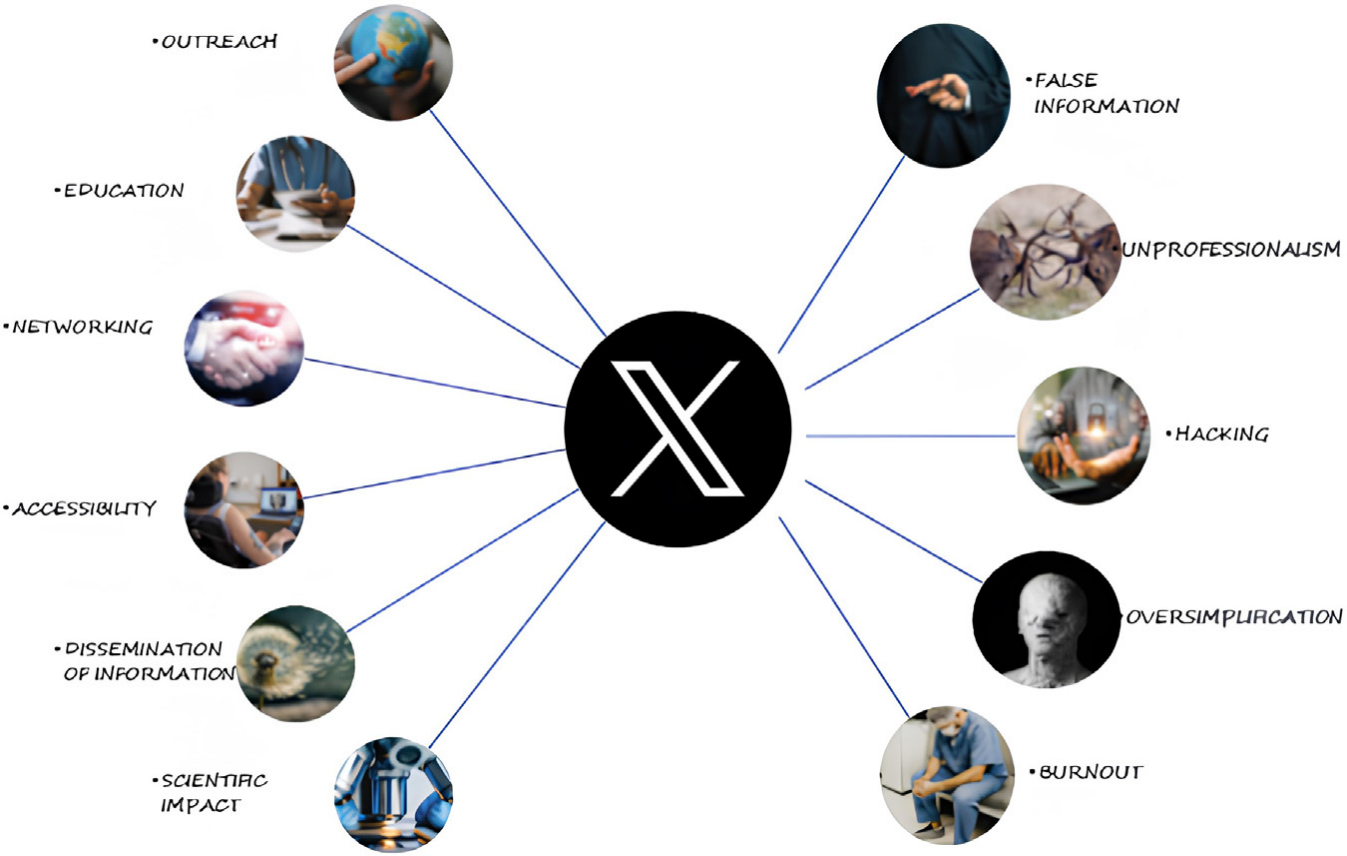
**Benefits and risks of Twitter (X) in cardiology.**
